# Supplementary material for: Modeling glioblastoma heterogeneity as a dynamic network of cell states
Source: Mol Syst Biol. 2021 Sep 16;17(9):e10105. doi: 10.15252/msb.202010105 (PMC8444284; doi:10.15252/msb.202010105)
Supplement: Supplementary file 6 — Source Data for Figure 5 [file MSB-17-e10105-s004.zip › Figure5A_sourcedata/GSEA_3017/hallmarks_stateC.GseaPreranked.1621934520804/HALLMARK_GLYCOLYSIS.html]

Details for gene set HALLMARK\_GLYCOLYSIS[GSEA]

|  || Dataset | state13017 |
| Phenotype | NoPhenotypeAvailable |
| Upregulated in class | na\_neg |
| GeneSet | HALLMARK\_GLYCOLYSIS |
| Enrichment Score (ES) | -0.4001867 |
| Normalized Enrichment Score (NES) | -1.5468706 |
| Nominal p-value | 0.0592 |
| FDR q-value | 0.07488564 |
| FWER p-Value | 0.505 |
Table: GSEA Results Summary

  

Fig 1: Enrichment plot: HALLMARK\_GLYCOLYSIS      
 Profile of the Running ES Score & Positions of GeneSet Members on the Rank Ordered List

  

| PROBE | GENE SYMBOL | GENE\_TITLE | RANK IN GENE LIST | RANK METRIC SCORE | RUNNING ES | CORE ENRICHMENT || 1 | NASP |  |  | 75 | 0.371 | -0.0677 | No |
| 2 | STMN1 |  |  | 85 | 0.357 | -0.0242 | No |
| 3 | CDK1 |  |  | 191 | 0.255 | -0.1627 | No |
| 4 | PYGB |  |  | 198 | -0.251 | -0.1316 | No |
| 5 | STC1 |  |  | 210 | -0.255 | -0.1082 | No |
| 6 | PGAM1 |  |  | 218 | -0.258 | -0.0776 | No |
| 7 | CHST2 |  |  | 350 | -0.297 | -0.2539 | No |
| 8 | VEGFA |  |  | 436 | -0.345 | -0.3432 | Yes |
| 9 | MIF |  |  | 442 | -0.347 | -0.2944 | Yes |
| 10 | HSPA5 |  |  | 447 | -0.350 | -0.2434 | Yes |
| 11 | LDHA |  |  | 498 | -0.412 | -0.2613 | Yes |
| 12 | NT5E |  |  | 500 | -0.416 | -0.1941 | Yes |
| 13 | IRS2 |  |  | 516 | -0.444 | -0.1465 | Yes |
| 14 | IGFBP3 |  |  | 538 | -0.493 | -0.1012 | Yes |
| 15 | GPC1 |  |  | 555 | -0.536 | -0.0401 | Yes |
| 16 | CD44 |  |  | 575 | -0.659 | 0.0361 | Yes |
Table: GSEA details [plain text format]

  

Fig 2: HALLMARK\_GLYCOLYSIS: Random ES distribution      
 Gene set null distribution of ES for **HALLMARK\_GLYCOLYSIS**
